# Molecular and Clinical Characterization of a Novel Prognostic and Immunologic Biomarker FAM111A in Diffuse Lower-Grade Glioma

**DOI:** 10.3389/fonc.2020.573800

**Published:** 2020-10-26

**Authors:** Xiaoshuai Ji, Feng Ding, Jiajia Gao, Xiaoming Huang, Wenqing Liu, Yunda Wang, Qian Liu, Tao Xin

**Affiliations:** ^1^ Department of Neurosurgery, Shandong Qianfoshan Hospital, Cheeloo College of Medicine, Shandong University, Jinan, China; ^2^ Department of Neurosurgery, Shandong Provincial Hospital, Cheeloo College of Medicine, Shandong University, Jinan, China; ^3^ Department of Neurosurgery, The First Affiliated Hospital of Shandong First Medical University, Jinan, China; ^4^ Department of Histology and Embryology, School of Basic Medical Sciences, Cheeloo College of Medicine, Shandong University, Jinan, China; ^5^ Department of Neurosurgery, Jiangxi Provincial People's Hospital Affiliated to Nanchang University, Nanchang, Jiangxi, China

**Keywords:** ****prognosis, tumor environment, immune response, lower grade glioma, immune checkpoint

## Abstract

**Background:**

Family with sequence similarity 111 member A (FAM111A), as a replication factor required for proliferating cell nuclear antigen (PCNA) loading, has been demonstrated a possible association with carcinogenesis. However, the role of FAM111A in lower-grade glioma (LGG) remains unclear. We aim at investigating the expression and function of FAM111A in lower-grade glioma at the molecular and clinical levels.

**Methods:**

In total, 711 lower-grade glioma samples were analyzed in our research, including 182 RNA-seq data from the Chinese Glioma Genome Atlas (CGGA) dataset and 529 RNA-seq data from The cancer Genome Atlas (TCGA) dataset. R language and the GraphPad software were used for the majority of statistical analysis and graphical work.

**Results:**

FAM111A expression was overexpressed in WHO grade III and IDH-wildtype lower-grade glioma. FAM111A was significantly downregulated in the IDHmut-Codel molecular subtype. Univariate and multivariate Cox analysis demonstrated that FAM111A was an independent prognostic factor in LGG patients. Functional characterization of FAM111A revealed that it was associated with inflammatory response and immune response to tumor cells. FAM111A could also act as an indicator of the stromal and immune population, especially for monocytic lineage, myeloid dendritic cells and fibroblasts. It was positively correlated with macrophages, especially the M2 macrophage cells. Furthermore, FAM111A revealed predictive value for the immune subtypes and immune checkpoint blockade therapy.

**Conclusion:**

FAM111A expression was closely related to the malignant phenotype, molecular pathology and immune response of lower-grade glioma. It might be a promising target for LGG immunotherapeutic strategies.

## Introduction

Gliomas, known for their heterogeneity and rapid clinical progression (1), are the most common and malignant brain tumor of the central nervous system in adults, which originates from the glial cells (2). Gliomas are generally divided into four grades (i.e., WHO grade I, II, III, and IV) based on the 2007 World Health Organization (WHO) classification of the central nervous system tumors (3). The term ‘diffuse lower-grade glioma’ (LGGs) refers to Grade II and III gliomas. In contrast to Grade IV gliomas-glioblastomas, LGGs tend to indicate a more favorable prognosis. However, majorities of LGGs advance to high-grade secondary aggressive gliomas and finally end with death (4, 5). Despite the advancements in surgical resection, adjuvant radiotherapy and chemotherapy, their prognosis is still poor.

The tumor microenvironment (TME), which is comprised of extracellular matrix, fibroblasts, vascular cells, neurons and immune cell, functions as a fundamental regulator of cancer occurrence, progression and invasion in primary and metastatic brain malignancies (6). Among infiltrating immune cells, macrophages and microglia account for the vast majority in gliomas (7, 8). Tumor-associated macrophages (TAMs), which frequently exhibit an M2 phenotype, have crucial bidirectional cross-talk with cancer cells. Brain tumor cells recruit TAMs by releasing cytokines and chemoattractant, and in turn TAM-derived pro-tumorigenic and pro-survival factors IL6, TNF, IL-1β, and IL-23 support tumor angiogenesis and invasion (6, 9). In addition, other lymphoid and myeloid lineage cells are found in TME and become a part of brain tumor biology in unique ways (6).To data, we still poorly understand the biology and function of these infiltrating immune cells in TME. As a new therapeutic strategy, TME targeted therapy has become a research hotspot. For cancer immunotherapy targeting TME, several approaches are ongoing in preclinical and clinical studies as an alternative and integrated strategy for the treatment of gliomas, either through monotherapy or *via* rational combinations. Of them, CSF-1R inhibitor, dendritic cell vaccine, anti-CTLA-4, and anti-PD-1 are gaining vital clinical attention for treatment to reactivate adaptive and innate immune systems (10–12). TME is primarily decided by the tumor genomic landscape. Therefore, further studies are needed to analyze the key immune-related genes and the interactions between immune cells and gliomas, which will contribute to deep discovery of underlying molecular mechanisms and novel strategies to improve efficacy of immunotherapies.

Family with sequence similarity 111 member A (FAM111A), also known as KCS2 and GCLEB, is a cell-cycle regulating and chromatin-associated protein-coding gene. Reduced gene expression of FAM111A leads to DNA replication defect, that applies to the replication of the Simian Virus 40 (SV40), indicating that it has antiviral properties (13). It contains a PCNA-interacting peptide (PIP) box and the carboxyl-terminal half, which are homologous to trypsin-like peptidases, and may interact with proliferating cell nuclear antigen (PCNA) (14). PCNA is overexpressed on the surface of cancer cells, and act as an immune checkpoint for NK-cell (15). The close relationship between FAM111A and PCNA in non-cancerous diseases suggests its potential function in immune responses. Additionally, several lines of evidence revealed correlations between FAM111A and tumorigenesis in prostate cancer and cervical cancer (16, 17). However, the detail of FAM111A expression and its role in glioma are still unknown.

In this research, we assessed the expression patterns of FAM111A to determine its potential functions and prognostic values in LGG based on data from the Chinese Gliomas Genome Atlas (CGGA) and The Cancer Genome Atlas (TCGA) datasets. To our best knowledge, this is the first comprehensive study exploring the role of FAM111A in LGG on a large-scale analysis. In particular, we found that FAM111A took part in various functional aspects of the immune system and may play as a new potential bio-target for immune therapy.

## Materials and Methods

### Patients and Data Collection

We downloaded 529 RNA-Seq gene expression profiles and clinical data of LGG patient samples from TCGA (http://cancergenome.nih.gov) databases. The molecular classification (the IDH and 1p/19q-based molecular subtypes) were obtained in the Merged Cohort of LGG and GBM (TCGA, Cell) from the cBioPortal for Cancer Genomics (http://cbioportal.org) (18, 19). We obtained the mRNA expression and clinical data of 182 LGG patient samples at the mRNAseq_325 Dataset from CGGA (http://www.cgga.org.cn/) databases (20, 21). To further identify the protein expression of FAM111A in LGG, a total of 8 LGG samples (4 each for grade II and grade III) were selected from the Department of Neurosurgery at Provincial Hospital. The research was approved by Shandong University Ethics Committee. We also got the tissue chips G6042-5 from Wuhan Servicebio Technology Co. Ltd, which included 2 WHO grade I, 16 WHO grade II, and 14 WHO grade III glioma patients samples (**Supplementary Table 1**).

### ONCOMINE Analysis

ONCOMINE gene expression array datasets (www.oncomine.org), an online cancer microarray database, was used to analyze the mRNA expression levels of FAM111A in different cancer types and glioma tissues. The cut-off of p value and fold change were defined as 0.01 and 1.5, respectively. It includes Sun Brain, French Brain, and Bredel Brain in glioma datasets.

### Immune Cell Infiltration Analysis

The ESTIMATE and MCP analysis was used to evaluate the relationship between FAM111A expression and tumor purity as well as the presence of infiltrating stromal/immune cells. CIBERSORT was utilized to evaluate the Macrophage and the Macrophage M2 proportion at the ‘Immune estimation’ part of the online website ‘TIMER 2.0’ (22, 23). CIBERSORT is an analytical tool developed by Newman et al., which provides an estimation of the population abundances of tissue-infiltrating cell types using gene expression data.

### Immunohistochemistry (IHC)

Paraffin-embedded lower-grade glioma tissue blocks were cut into 4 μm sections and then analyzed by immunohistochemistry (IHC). The staining intensity was classified as four grades: 0 (negative), 1 (weak), 2 (moderate) and 3 (strong). FAM111A protein expression (semi-quantitative scoring by using H-score system): H-score = (percentage of cells with weak intensity staining ×1) + (percentage of cells with moderate intensity staining ×2) + (percentage of cells with strong intensity staining ×3). The scoring was automatically measured by Quant Center software and the H-score ranges from 0 to 300. The scores of duplicate specimens were averaged. CD163, CD206, CD276, TIM-3 protein expression was analyzed by using the Indica Labs-Multiplex IHC v2.2.0 analysis software to quantify the number of positive cells and the total number of cells in the target area of each slice to determine the positive rate. IHC: FAM111A (1:20); CD163 (1:500); CD206 (1:1,000); CD276 (1:100); TIM-3 (1:100) Zen Bioscience, China.

### Quantitative Real-Time PCR (qRT-PCR)

Total RNA was isolated from the tissue samples using Trizol reagent (Invitrogen, USA). QRT-PCR was performed using a Real-Time PCR System (Bio-rad, USA) with a SYBR Green PCR kit (TransGen biotech, China) following the manufacturer’s instructions. Comparative quantification was evaluated through the 2^−ΔCt^ method and GAPDH was as the endogenous control. The primer sequences were as follows:

FAM111A, 5’-CTTCACAAAAAGGGGCGCAA -3’ (forward) and 5’ ATCAACTGGCTGGGTGCTTT-3’ (reverse);

JAK2, 5’-TCTGGTGCCTTTGAAGACCG -3’ (forward) and 5’- GCACATCTCCACACTCCCAA-3’ (reverse);

STAT3, 5’- ACGAAGGGTACATCATGGGC-3’ (forward) and 5’- CTGGATCTGGGTCTTACCGC-3’ (reverse);

NFKB1, 5’-ATGTGGGACCAGCAAAGGTT -3’ (forward) and 5’- CACCATGTCCTTGGGTCCAG-3’ (reverse).

### Statistical Analysis

The statistical software R (version 3.6.3), IBM SPSS Statistics 25 (version 25.0.0.1), GraphPad Prism 8 software (version 8.3.0), Adobe Illustrator CC 2018 (version 22.0.0) were used for the statistical analysis and generation of figures. The value of FAM111A expression level above the median value (7.5 for CGGA, 2.6 for TCGA) were defined as high-expression group and the value of FAM111A expression level below the median was defined as the low-expression group. The GSEA molecular signatures dataset (MSigDB) hallmark gene sets were used to perform pathway analysis (24). The enrichment status estimates of inflammatory response-associated metagenes were obtained using the GSVA package. Correlograms and circus plots were performed using the “circlize” package, and the R packages “ImmuneSubtypeClassifier” was used to identify six immune subtypes, which describe a categorization of tumor-immune status. Other R packages, “ggplot2”, “plotROC”, and “xgboost” were also applied for visualizing the results of data analysis. All statistical tests were two-sided and p < 0.05 was considered a significant difference.

## Results

### The mRNA Expression of FAM111A in LGGs

**Figure 1:**
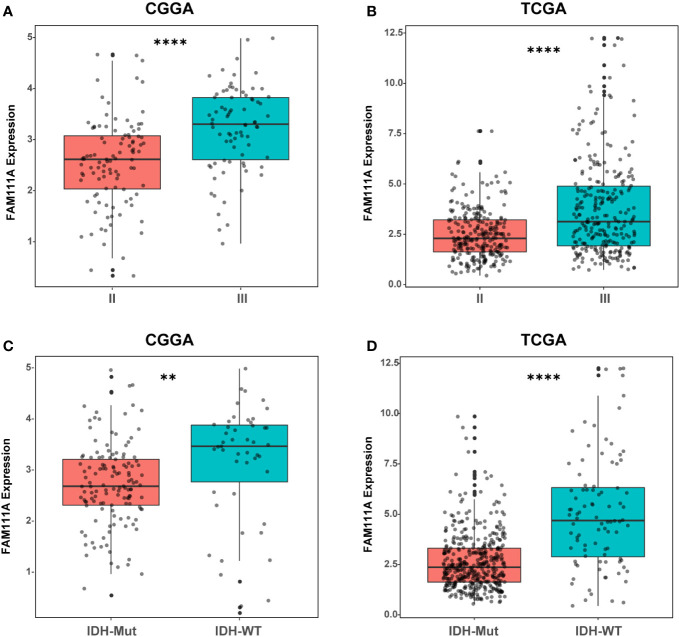
The mRNA expression of FAM111A in lower-grade glioma. **(A, B)** FAM111A was significantly increased in WHO grade III gliomas in Chinese Glioma Genome Atlas (CGGA) and The Cancer Genome Atlas (TCGA) dataset. **(C, D)** FAM111A was significantly increased in isocitrate dehydrogenase (IDH)-wildtype lower-grade glioma (LGG) in CGGA and TCGA dataset. *P < 0.05, **P< 0.01, ***P < 0.001, ****P < 0.0001.

**Figure 2:**
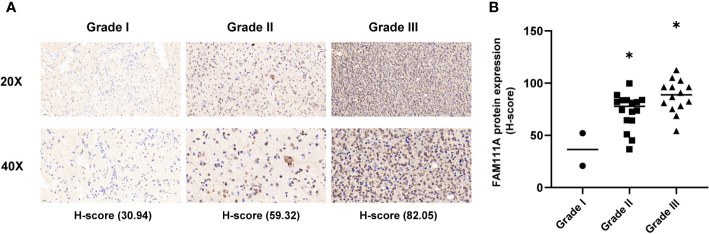
The protein expression of FAM111A in lower-grade glioma (IHC). **(A)** upper, × 20; lower, × 40. **(B)** The staining of FAM111A was scored based on the H-score system. FAM111A protein expression was higher in high-grade lower-grade glioma (LGG). *P < 0.05, **P< 0.01, ***P < 0.001.

Based on the data from Oncomine databases, the mRNA expression of FAM111A in LGGs were significantly higher in comparison with that in normal brain tissues (**Supplementary Figures 1A–G**). To characterize the expression pattern of FAM111A in LGGs, we investigated the RNA-Sequencing data based on WHO glioma grades from the CGGA and TCGA datasets. Compared to WHO grade II gliomas in the CGGA dataset, WHO grade III gliomas showed a higher mRNA expression of FAM111A (**Figure 1A**). Consistent results were validated using the TCGA RNA-seq data (**Figure 1B**). Taken together, these results suggest that FAM111A was significantly up-regulated in WHO grade III gliomas. Several reports have shown that isocitrate dehydrogenase (IDH) mutation plays a crucial role in the development and progression of glioma (25, 26). We found that FAM111A was indeed highly elevated in IDH wild-type gliomas in both of the CGGA and TCGA data sets (**Figures 1C, D**). What’ more, we performed the immunohistochemistry (IHC) to explore the relation between tumor grade, and FAM111A protein expression. The results showed that the FAM111A protein expression was higher in high-grade gliomas (**Figures 2A, B**).

### FAM111A Is a Potential Marker for the IDHMut-Codel Molecular Subtype in LGGs

**Figure 3:**
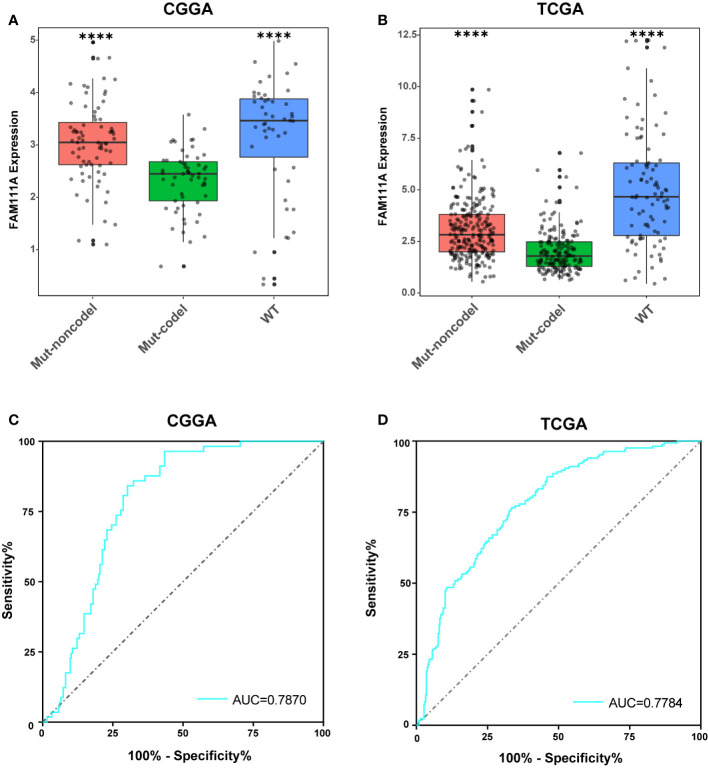
FAM111A expression pattern in different molecular subtypes. **(A, B)** FAM111A was significantly downregulated in the IDHmut-Codel molecular subtype in CGGA and TCGA dataset. **(C, D)** The predictive value of FAM111A expression in Chinese Glioma Genome Atlas (CGGA) and The Cancer Genome Atlas (TCGA) dataset by ROC curve analysis. *P < 0.05, **P < 0.01, ***P < 0.001, ****P < 0.0001.

According to the phenotypes and genotypes (27, 28), based on mutation of the IDH1 and IDH2 genes and codeletion of chromosomes 1p and 19q, lower-grade gliomas can be classified as three molecular subtypes: IDH wild type (IDHwt), IDH mutant with 1p/19q codeletion (IDHmut-Codel) or IDH mutant with no 1p/19q codeletion (IDHmut-Noncodel) (29). To seek the molecular expression pattern of FAM111A, we evaluated the expression of FAM111A in the three molecular subtypes of LGGs. Based on the CGGA and TCGA RNA-seq data, FAM111A was significantly downregulated in the IDHmut-Codel molecular subtype compared to the other molecular subtypes (**Figures 3A, B**). To further confirm the findings above, ROC curves for FAM111A expression and IDHmut-Codel molecular subtype of LGGs were performed. The results showed that area under the curve (AUC) were 78.70% and 77.84% in CGGA and TCGA data set, respectively, indicating that FAM111A is a potential marker for the IDHMut-codel molecular subtype in LGGs (**Figures 3C, D**).

### High Level of FAM111A Predicted Worse Survival in LGGs

**Figure 4:**
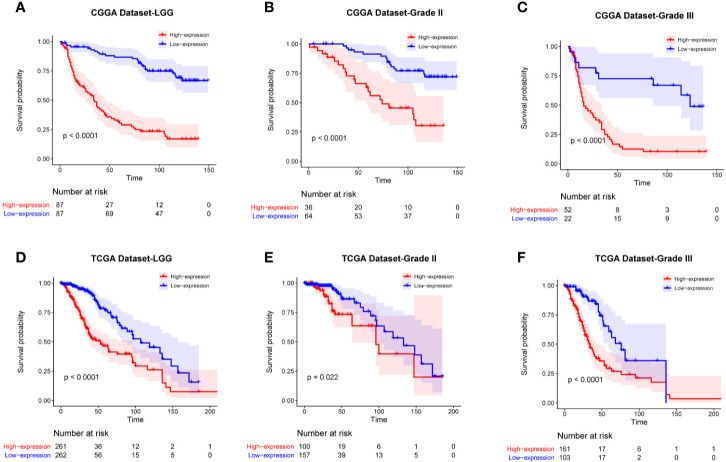
Kaplan-Meier survival curves comparing the high and low expressions of FAM111A in lower-grade glioma. High expression of FAM111A predicted worse outcome in lower-grade glioma patients **(A, D)**, WHO grade II **(B, E)**, and WHO grade III **(C, F)** gliomas patients in Chinese Glioma Genome Atlas (CGGA) and The Cancer Genome Atlas (TCGA) dataset.

To determine the prognostic value of FAM111A, the survival time of all the 697 LGG patients from the CGGA and TCGA RNA-seq was analyzed by Kaplan-Meier method. As shown in **Figure 3**, high expression of FAM111A predicted a remarkably shorter overall survival (OS) both in CGGA (**Figures 4A–C**) and in TCGA datasets (**Figures 4D–F**) in LGGs, which included WHO grade II and WHO grade III gliomas. Then, we used univariate and multivariate Cox regression to determine whether FAM111A expression acted as an independent prognostic factor (**Table 1**). Interestingly, in univariate analysis of the two databases, FAM111A along with age, grade, and IDH status remarkably predicted the OS in LGG. In multivariate regression, it also revealed that the expression of FAM111A was an independent prognosticator after adjusting for all the clinical factors in the table. These findings evidently suggested that FAM111A predicted poor prognosis in LGGs.

**Table 1:** Univariate and multivariate Cox analysis of clinic-pathologic characteristics in lower-grade gliomas based on Chinese Glioma Genome Atlas (CGGA) and The Cancer Genome Atlas (TCGA) datasets.

Datasets	Characteristic	HR	95%CI	P		HR	95%CI	P
**CGGA**	Univariate				Multivariate			
	Age	3.187	1.632-6.224	0.001	Age	1.375	0.675-2.800	0.381
	Gender	0.612	0.404-0.928	0.021	Gender	0.627	0.408-0.965	0.034
	Grade	3.745	2.435-5.759	0.000	Grade	2.706	1.729-4.234	0.000
	IDH-Mut	0.385	0.247-0.600	0.000	IDH-Mut	0.675	0.420-1.087	0.106
	FAM111A	5.468	3.383-8.836	0.000	FAM111A	4.211	2.547-6.961	0.000
**TCGA**	Univariate				Multivariate			
	Age	4.848	3.205-7.334	0.000	Age	3.513	2.014-4.937	0.000
	Gender	1.144	0.814-1.606	0.438	Gender	1.236	0.851-1.794	0.266
	Grade	3.296	2.277-4.771	0.000	Grade	2.159	1.431-3.256	0.000
	IDH-Mut	0.182	0.126-0.261	0.000	IDH-Mut	0.323	0.210-0.495	0.000
	FAM111A	2.946	2.045-4.243	0.000	FAM111A	1.823	1.204-2.760	0.005

### FAM111A Was Closely Associated With Immune Functions in LGGs

**Figure 5:**
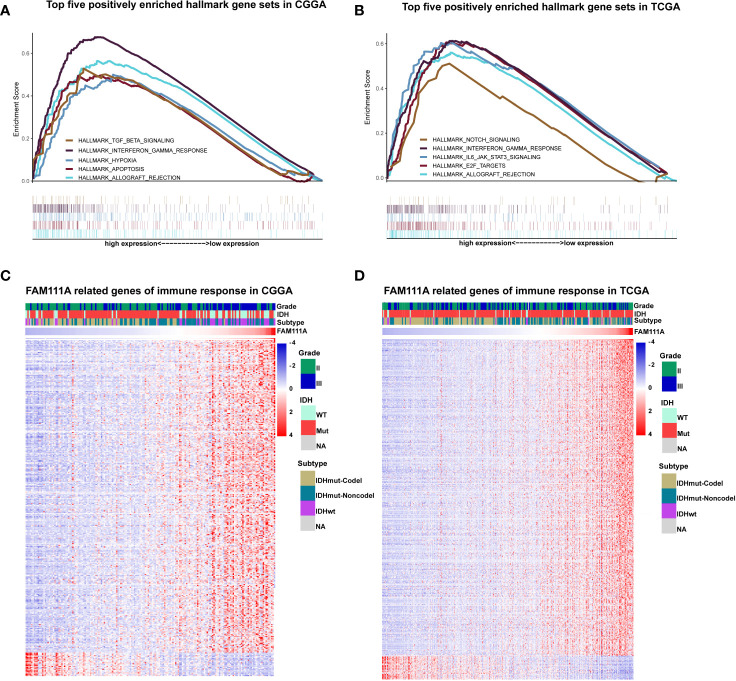
FAM111A was closely associated with immune functions in lower-grade glioma. **(A, B)** Enrichment plots of the top five enriched signaling pathways in FAM111A-high expression phenotype from gene set enrichment analysis (GSEA). **(C, D)** Most immune response related genes were significantly positively correlated with FAM111A expression, while a few genes were negatively correlated with FAM111A expression in Chinese Glioma Genome Atlas (CGGA) and The Cancer Genome Atlas (TCGA) dataset.

Due to the vital role of FAM111A in LGG progression and prognosis, we aim to explore the function of FAM111A in both of the CGGA and TCGA databases. The specifically enriched signaling pathway in FAM111A-high expression samples could be substitutable targets for FAM111A. Based on the FAM111A median expression, samples were divided into two groups. We subsequently performed gene set enrichment analysis (GSEA) using the MSigDB hallmark gene sets to obtain significantly different expressing genes between these two groups (24). Immune response-related gene sets were highly positively enriched when FAM111A-high expression with FAM111A-low expression samples were compared (**Figures 5A, B** **;** **Supplementary Figures 2A, B**). Among the top five significantly enriched hallmark gene sets, both datasets contained “Hallmark interferon gamma response”. We then used the real time qRT-PCR to detect the mRNA expression of 3 “Hallmark interferon gamma response” related genes (JAK2, STAT3 and NFKB1) from 8 LGG tissue samples, and divided them into the high expression group and the low expression group according to the FAM111A mRNA expression. Results showed that the mRNA expression of JAK2 and STAT3 were significantly enriched in the high expression group (**Supplementary Figures 3A–C**).

To further identify the role of FAM111A in immune response in LGGs, we downloaded immune-associated gene sets from the AmiGO 2 Web portal (http://amigo.geneontology.org/amigo). Genes most relevant to FAM111A (|R| > 0.4 and p < 0.05) were selected for heat-map drawing. Among the 369 selected genes in the CGGA datasets, 343 immune-related genes were significantly positively correlated with FAM111A expression. On the other hand, a significantly positive correlation also existed between FAM111A expression and 399 genes out of the 429 genes selected in the TCGA datasets (**Figures 5C, D**). A detailed list of these genes was presented in **Supplementary Table S2, 3**. Overall, these results indicated that FAM111A might be closely associated with immune responses in LGGs.

### FAM111A-Related Inflammatory Activities

**Figure 6:**
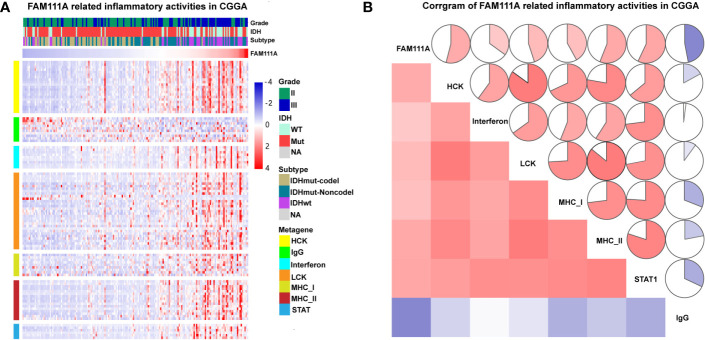
FAM111A-related inflammatory activities in lower-grade glioma. **(A)** The heatmap of the relationship between FAM111A and seven inflammatory metagenes in Chinese Glioma Genome Atlas (CGGA) dataset. **(B)** Correlogram showed the correlation between FAM111A and seven inflammatory metagenes in CGGA dataset.

Inflammation not only exerts cancer-promoting effects, but also plays an important role in the host immune response to tumors as well as cancer immunotherapy (30). To determine which types of inflammatory activities were related to FAM111A, we altogether chose seven metagenes of 104 genes (31), which were related to different types of inflammation and immune responses (**Supplementary Table S4**). Gene Sets Variation Analysis (GSVA) were subsequently performed to convert the expression data of these metagenes into enrichment scores. The results, as shown in **Figure 5**, indicated that FAM111A was positively associated with HCK, LCK, MHC‐I, MHC II, and STAT1, but negatively correlated with IgG, which mainly refers to the activities of B-lymphocytes both in the CGGA and TCGA datasets (**Figures 6A, B**, **Supplementary Figure 3D, F**).

### Association of FAM111A and Immune Cell Populations in the TME

**Figure 7:**
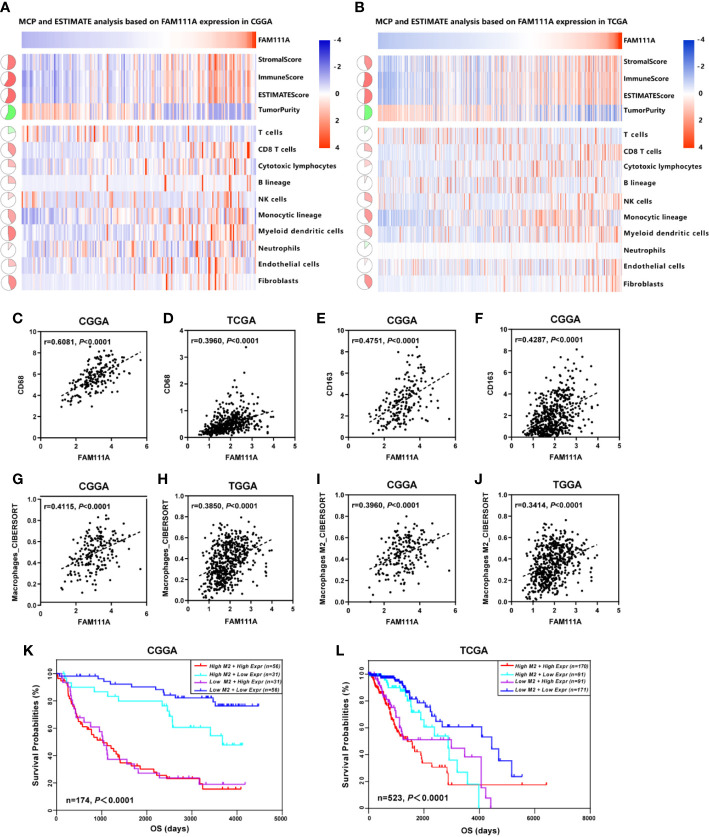
FAM111A-related immune population in the TME. **(A, B)** FAM111A was positively related to the immune scores and immune infiltrating cell population in Chinese Glioma Genome Atlas (CGGA) and The Cancer Genome Atlas (TCGA) dataset. FAM111A was significantly correlated with CD68 **(C, D)** and CD163 **(E, F)** in both datasets. Abundance of macrophage and M2 macrophage immune cell infiltration was quantified based on mRNA expression data with TIMER and CIBERSORT. Results showed significant correlations between FAM111A expression and macrophage cells **(G, H)** as well as the M2 macrophage cells **(I, J)**. Survival was stratified by a combined analysis of FAM111A expression and M2 macrophage infiltration level in CGGA and TCGA cohort. Survival based on high/low FAM111A expression and high/low infiltration levels of M2 macrophage in CGGA **(K)** and TCGA **(L)** dataset.

The MCP counter and ESTIMATE methods were used to investigate the relationship between FAM111A expression and immune cell infiltration. Both methods indicated that FAM111A was strongly related to the immune score and immune infiltrating cell population, especially for CD8 T cells, monocytic lineage, myeloid dendritic cells, and fibroblasts (**Figures 7A, B**).

It is acknowledged that M2 macrophages plays a vital role in the immunosuppression, tumor progression and metastasis of gliomas (32). Therefore, we aimed to explore whether expression of FAM111A was associated with the macrophages, especially the M2 macrophages in the TME. Consistent with our hypothesis, positive correlations was seen between CD68 and FAM111A (**Figures 7C, D**), as well as between CD163 and FAM111A (**Figures 7E, F**), a marker of macrophage and M2 macrophage respectively. The results of CIBERSORT were also in accordance with the above-mentioned findings (**Figures 7G–J**). Moreover, we performed IHC staining of M2 macrophage markers including CD163 and CD206 in LGG tissues, and results showed that the protein expression of these markers was higher in high FAM111A expression LGG samples (**Supplementary Figure 4A**).

Although FAM111A expression was associated with the M2 macrophages, some samples with high expression of FAM111A had low levels of M2 macrophage infiltrations. Therefore, we took a step further to investigate the survival of patients with high or low infiltration of M2 macrophages stratified by FAM111A expression (**Figures 7K, L**). Interestingly, patients with the best survival had low-expression of FAM111A together with low infiltration of M2 macrophages, whereas patients with high-expression of FAM111A as well as elevated level of M2 macrophages infiltration had the worst survival. These results suggested that the combined analysis of FAM111A and levels of M2 macrophages infiltration could yield different subtypes of LGGs; specifically, high levels of M2 macrophages infiltration in the absence of FAM111A expression might herald improved survival in LGG patients.

### FAM111A Expression in Different Immune Subtypes

**Figure 8:**
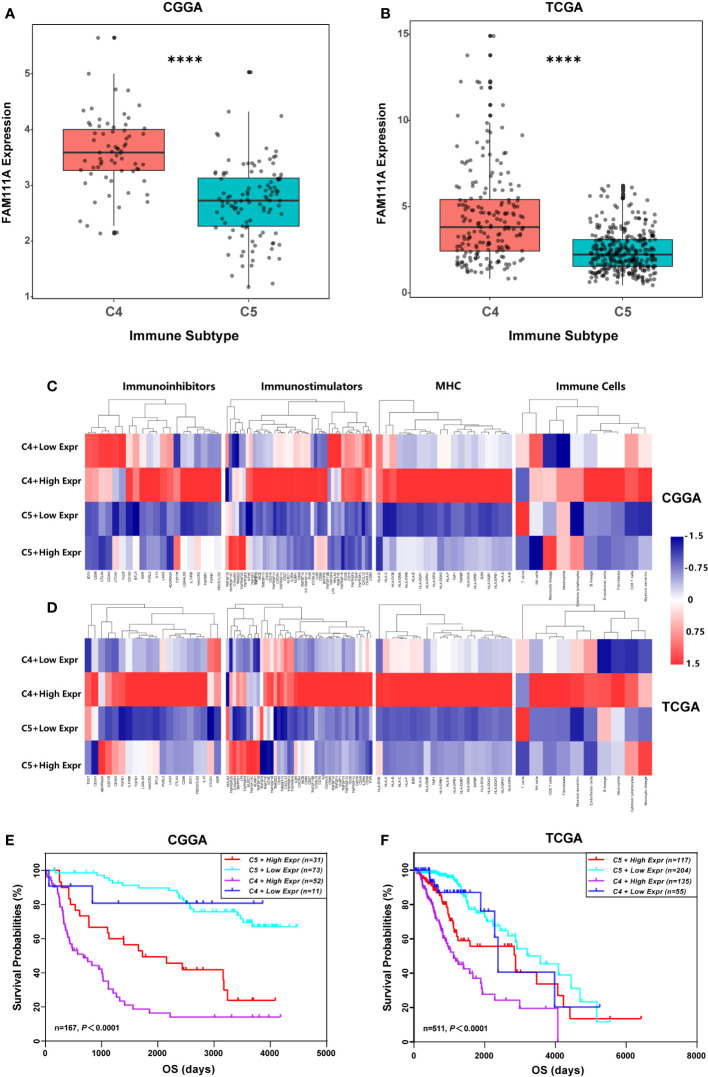
FAM111A expression in the immune subtypes. **(A, B)** FAM111A expression was significantly overexpressed in the C4 subtype in Chinese Glioma Genome Atlas (CGGA) and The Cancer Genome Atlas (TCGA) dataset. **(C, D)** C4 or C5 stratified by FAM111A expression exhibited distinct expression patterns of immunoregulatory genes and compositions of immune cells. Average z-score of each immune-regulatory gene and immune cell was obtained. **(E, F)** Kaplan–Meier survival curves of OS among four patient groups stratified by the FAM111A expression and the immune subtype. C4: the Immunologically Quiet subtype, C5: the Lymphocyte Depleted subtype. *P < 0.05, **P< 0.01, ***P < 0.001, ****P < 0.0001.

Recently Thorsson et al. identified six immune subtypes that define distinct immune response patterns influencing prognosis based on immunogenomics analyses of over 10,000 tumors (33). To examine the relationship between FAM111A expression and the six immune subtypes, ImmuneSubtypeClassifier was used. The results showed that LGGs consisted most of C4 (Immunologically Quiet) and C5 (Lymphocyte Depleted) subtypes (**Supplementary Figure 5A, B**), which was in line with Thorsson’s findings. FAM111A was significantly overexpressed in C4 (**Figures 8A, B**) that had the worse prognosis than C5 in both datasets (**Supplementary Figure 5C, D**). Results of ROC curve analysis also showed that the AUC was 84.01% and 75.11% in the CGGA and TCGA RNA-seq datasets, respectively (**Supplementary Figure 5E, F**). To explore the significance of FAM111A expression in different immune subtypes, we further compared the immune-regulatory gene expression profiles and immune-cell compositions of C4 or C5 stratified by FAM111A expression (34). Results showed that the four groups had a distinct immune pattern, especially for C4 with high FAM111A expression and C5 with low FAM111A expression (**Figures 8C, D**). A detailed list of immune-regulatory genes was presented in **Supplementary Table S5**. Moreover, in comparison to C4 with low-expression FAM111A, C4 with high-expression of FAM111A had worse prognosis. Consistently, C5 with high-expression of FAM111A predicted worse survival compared to C5 with low levels of FAM111A in LGGs (**Figures 8E, F**). These findings suggested that differential expression of FAM111A might define distinct response patterns and further subdivide the immune subtypes.

### Relationships Between FAM111A Expression and Immune Checkpoints

**Figure 9:**
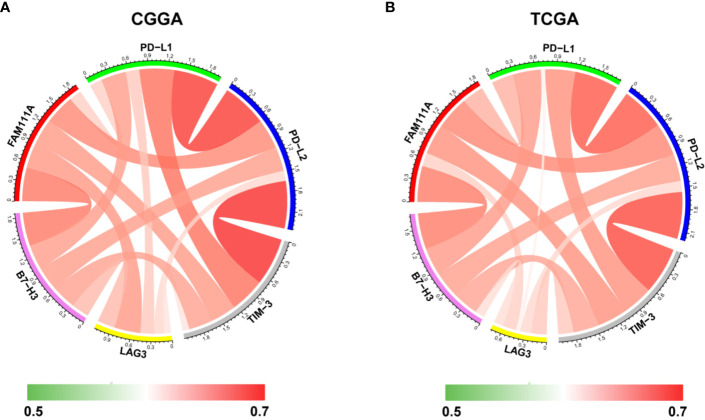
Relationships between FAM111A expression and immune checkpoint markers. Correlation between FAM111A and immune checkpoints (PD-L1, PD-L2, TIM-3, LAG3, B7-H3) was analyzed by Pearson Correlation Coefficient and visualized using circus plots in Chinese Glioma Genome Atlas (CGGA) **(A)** and The Chinese Genome Atlas (TCGA) **(B)** dataset.

Immune checkpoints play a crucial role in tumor immunosuppression. Therefore, we analyzed the relationship between expression of FAM111A and the immune checkpoint-related genes by Pearson correlation analysis in LGGs, including CD274 (PD‐L1), PD-L2 (PDCD1LG2), HAVCR2 (TIM‐3), LAG3 and B7-H3 (CD276). Results showed that FAM111A was mostly relevant to PD-L2, B7-H3, and TIM-3 in CGGA and TCGA datasets (**Figures 9A, B**). The results of IHC also revealed that B7-H3 and TIM-3 were highly expressed in high FAM111A-expression LGG samples compared with the negative ones (**Supplementary Figure 4B**), evidently suggesting possible synergistic effects of FAM11A with these checkpoint genes.

## Discussion

Glioma is a severe malignant brain tumor that affects human health severely. WHO grades II and III gliomas are categorized as LGGs that naturally undergo anaplastic conversion or dedifferentiation into glioblastoma on average of about 4–5 years following diagnosis. Despite aggressive treatment including surgery, radiotherapy, chemotherapy and targeted agents, glioma still has a dismal prognosis. (35). Tumor immunotherapies targeting TME, as a novel treatment strategy for glioma patients has shown a promising prospect. However, by increasing the activity of immune system, immune checkpoint blockade could cause immune-related adverse events, such as inflammation involved in the central nervous system (36). Thus, it is critical to explore the new key immune-related genes in order to achieve a comprehensive understanding of the tumor immune landscape, further improving efficacy of immunotherapies of gliomas.

Recent studies demonstrated that FAM111A played an inhibitory role in viral DNA replication and might have the anti-viral effects (37), which suggested its potential function in immune responses. Based on data from the Oncomine database, FAM111A expression was remarkably upregulated in LGGs. When applied to the TCGA and CGGA RNA-seq datasets, FAM111A exhibited a significant association with glioma grades and IDH mutation status. The FAM111A protein expression was also higher in high-grade gliomas and IDH wild-type gliomas. We also found that FAM111A was significantly downregulated in the IDHmut-Codel molecular subtype, indicating that FAM111A was associated with higher malignant biologic processes. Additional, survival analysis was consistent with this finding. Furthermore, after adjusting for traditional clinical factors, FAM111A remained an independent prognostic factor.

In order to elucidate the biological functions of FAM111A in LGG, we performed GSEA analysis using the MSigDB hallmark gene sets. The immune response-related gene signatures, including “Hallmark interferon gamma response” were among the top positively enriched pathways in the FAM111A high-expression group. Interferon gamma (IFN-γ), a pleiotropic cytokine regarded as an important effector molecule of anti-tumor immunity, has been involved in promoting immunosuppressive TME through inducing immunosuppressive gene expression signature in cancer cells (PD-L1, PD-L2, CTLA-4, etc.) (38). IL-6/JAK/STAT3 and TGF-β signaling also participated in cancer inflammation and immunity (39). Furthermore, to determine the immune response that FAM111A was involved in, we explored the relationship between FAM111A and seven immune-related clusters. We found that FAM111A was positively correlated with T-cell and macrophages mediated immune responses, but negatively correlated with the B lymphocytes-related response. Characterization of immune subpopulation infiltration by MCP and ESTIMATE analysis indicated that FAM111A was significantly related to monocytic lineage, CD8 T cells, myeloid dendritic cells and fibroblasts. To make a step further, we carried out CIBERSORT analysis, and detected a positive relationship between FAM111A and M2 macrophages. Taken together, it is most likely that FAM111A played a critical role in the tumor immunity and may act as an immune suppressor in LGGs.

Six immune subtypes, from C1 to C6, characterize the gross immune response patterns of several heterogeneous tumors. LGG mainly consists of C4 (Lymphocyte Depleted) and C5 (Immunologically Quiet) subtypes. C5 exhibits fewer tumor-associated immune cells and better outcome, with the enriched CpG island methylator phenotype-high (CIMP-H), the 1p/19q codeletion and pilocytic astrocytoma-like (PA-like) as well as the IDH mutations. Nevertheless, the C4 subtype displays a more prominent macrophage signature, with low lymphocytic infiltrate and high M2 macrophage, which leads to an immunosuppressed TME and a poor outcome. Our results showed that FAM111A was overexpressed in the C4 subtype and roughly in accord with immune characteristics of the C4 subtype, which revealed that FAM111A possibly referred to a negative microenvironment. Moreover, conjoint analysis of FAM111A expression may identify a more detailed immunophenotype in LGGs. Interestingly, the C4 with high-expression FAM111A expression group was also related to the MHCs and immunostimulators, which might indicate the activation of the interferon gamma response pathway and a more complex relationship between FAM111A and tumor immunity in LGGs.

Immunomodulatory therapies targeting immune checkpoint molecules have revolutionized the treatment of solid tumor malignancies (40, 41). Due to the significance of ICB therapy, we evaluated the correlations between FAM111A and inhibitory checkpoint molecules. The results showed that FAM111A was tightly associated with PD-L2, TIM-3, and B7-H3, which was also confirmed by IHC. These results, taken together with previous observations, suggested that FAM111A was closely linked to an immunosuppressive phenotype and maybe a potential predictive marker for ICB response.

In conclusion, as far as we know, this is the first study to investigate the gene expression, clinical characteristics and biological functions of FAM111A on large-scale samples of LGGs. FAM111A revealed a significant association with immune response and an immunosuppressive tumor microenvironment in LGGs. These novel findings would provide a new perspective for cancer immunotherapy, enabling more precise and personalized glioma chemotherapeutic intervention in the future.

## Data Availability Statement

The original contributions presented in the study are included in the article/supplementary material. Further inquiries can be directed to the corresponding authors.

## Ethics Statement

The studies involving human participants were reviewed and approved by Shandong University Ethics Committee. The patients/participants provided their written informed consent to participate in this study.

## Author Contributions

XJ, FD, QL, and TX conceived the concept and designed this study. JG, XH, WL, and YW extracted the information from the databases. XJ and JG participated in data analysis and figure preparation. XJ wrote the manuscript. QL, TX, and FD reviewed the manuscript. All authors contributed to the article and approved the submitted version.

## Funding

This work was supported by National Natural Science Foundation of China (Grant No. 81972340), Shandong Provincial Natural Science Foundation (Grant No. ZR2016HM59), Science and Technology Project of Jinan City (Grant No. 201907048), Key Projects of Natural Science Foundation of Jiangxi Province (Grant No. 20192ACB20011).

## Conflict of Interest

The authors declare that the research was conducted in the absence of any commercial or financial relationships that could be construed as a potential conflict of interest.
