# Supplementary material for: Molecular and Clinical Characterization of a Novel Prognostic and Immunologic Biomarker FAM111A in Diffuse Lower-Grade Glioma
Source: Front Oncol. 2020 Oct 26;10:573800. doi: 10.3389/fonc.2020.573800 (PMC7649369; doi:10.3389/fonc.2020.573800)
Supplement: Supplementary file 1 [file DataSheet_1.pdf]

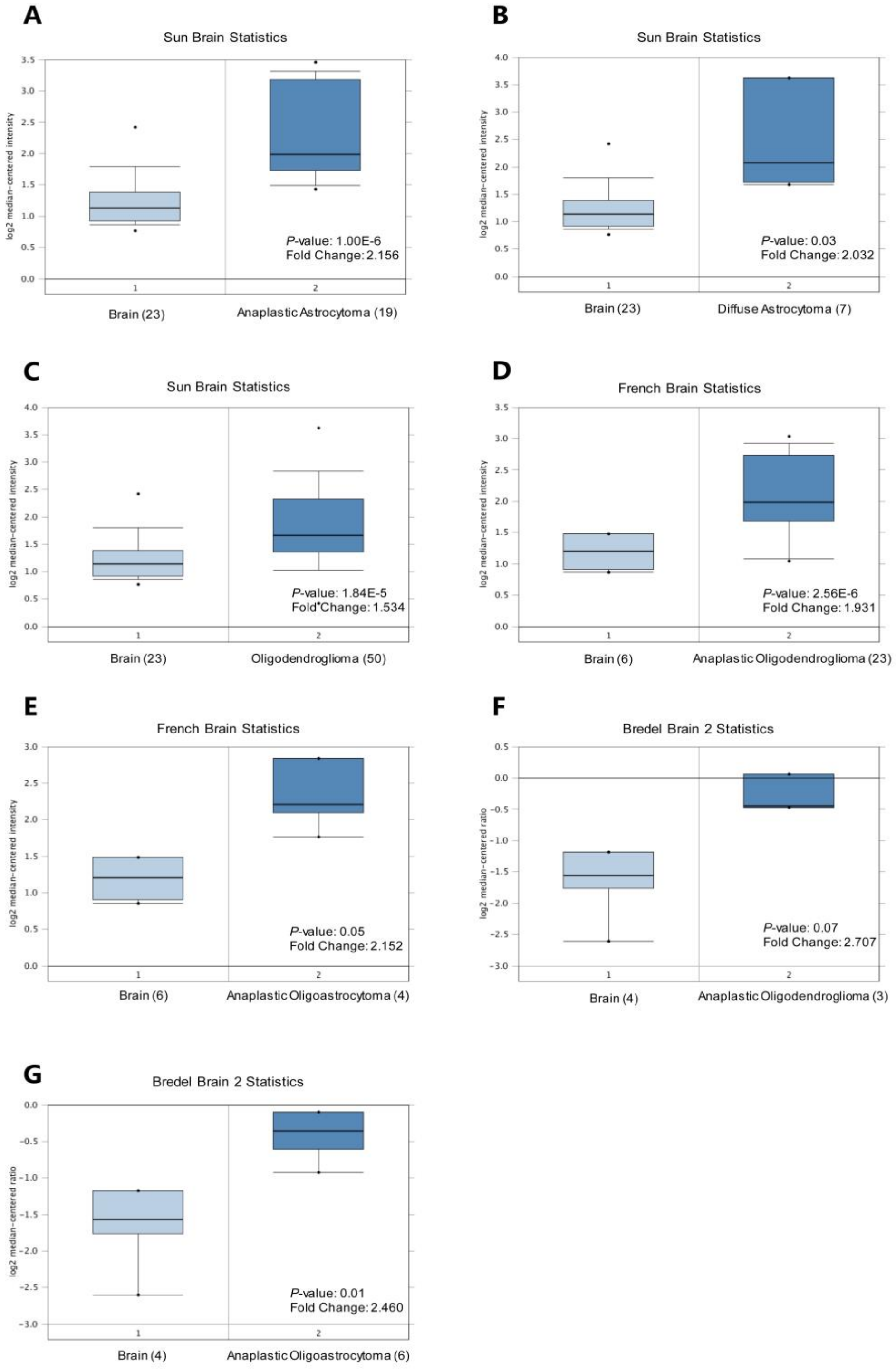

Supplementary Figure 1. Oncomine database analyses of FAM111A mRNA expression in lower-grade gliomas. (A-G) Levels of FAM111A mRNA expression were significantly higher in lower-grade glioma than in normal tissue.

A

(CGGA) FAM111A associated pathways

	Hallmark gene sets	Size	Normalized enrichment score	NOM p-value
1	HALLMARK_INTERFERON_GAMMA_RESPONSE	195	1.895828	0.0125
2	HALLMARK_APOPTOSIS	157	1.7621692	0.016260162
3	HALLMARK_ALLOGRAFT_REJECTION	182	1.7324585	0.030549899
4	HALLMARK_TGF_BETA_SIGNALING	54	1.7253009	0.013565891
5	HALLMARK_HYPOXIA	184	1.7215838	0.047904193
6	HALLMARK_IL6_JAK_STAT3_SIGNALIN	81	1.7213043	0.04024145
7	HALLMARK_INTERFERON_ALPHA_RESPONSE	95	1.7124549	0.05394191
8	HALLMARK_GLYCOLYSIS	190	1.710461	0.020491803
9	HALLMARK_TNFA_SIGNALING_VIA_NFKB	195	1.6942704	0.06090373
10	HALLMARK_E2F_TARGETS	194	1.6827588	0.049808428

B

(TCGA) FAM111A associated pathways

	Hallmark gene sets	Size	Normalized enrichment score	NOM p-value
1	HALLMARK_NOTCH_SIGNALING	32	1.8164387	0.003984064
2	HALLMARK_IL6_JAK_STAT3_SIGNALING	87	1.7787125	0.022222223
3	HALLMARK_INTERFERON_GAMMA_RESPONSE	198	1.7422824	0.036821704
4	HALLMARK_ALLOGRAFT_REJECTION	195	1.7062465	0.033797216
5	HALLMARK_E2F_TARGETS	195	1.6436566	0.05882353
6	HALLMARK_INTERFERON_ALPHA_RESPONSE	95	1.6231915	0.07156309
7	HALLMARK_IL2_STAT5_SIGNALING	195	1.6149842	0.02952756
8	HALLMARK_G2M_CHECKPOINT	189	1.5774671	0.084848486
9	HALLMARK_APOPTOSIS	159	1.522852	0.06339468
10	HALLMARK_PI3K_AKT_MTOR_SIGNALING	103	1.5037365	0.047808766

Supplementary Figure 2. Gene set enrichment analysis between FAM111A high- and low-expression samples. (A, B) GSEA positive result table showing the top ten enrichment terms of the hallmark gene sets from MSigDB in CGGA and TCGA datasets.

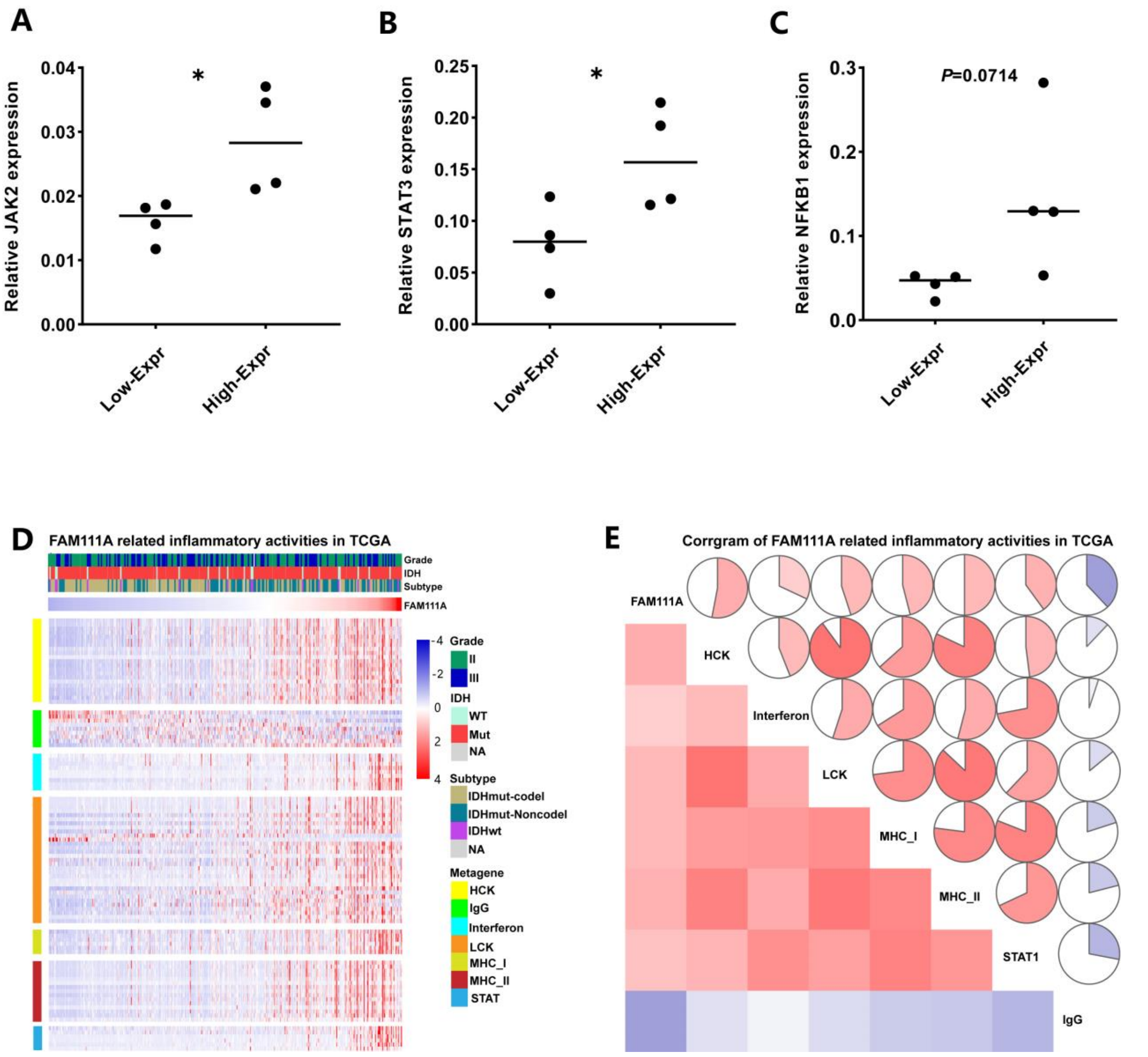

Supplementary Figure 3. (A, B, C) The mRNA expression of JAK2 and STAT3 were significantly enriched in the FAM111A high expression group. FAM111A, JAK2, STAT3 and NFKB1 expression level was normalized to that of GAPDH. (D) The heatmap of the relationship between FAM111A and seven inflammatory metagenes in TCGA dataset. (E) Correlogram showed the correlation between FAM111A and seven inflammatory metagenes in TCGA dataset. *P < 0.05, **P< 0.01, ***P < 0.001.

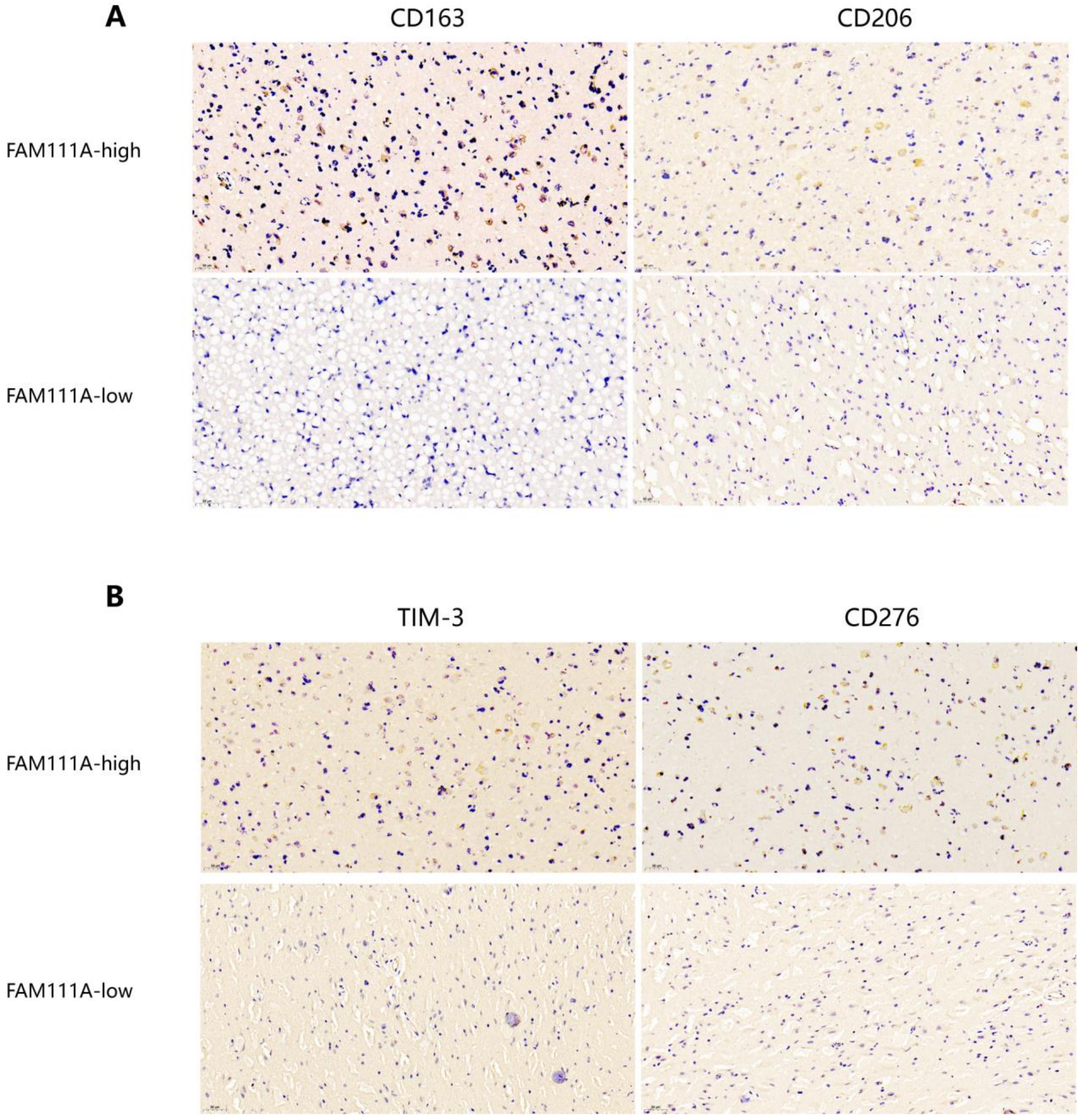

Supplementary Figure 4. Expression of CD162, CD206 (A), TIM-3 and CD276 (B) in FAM111A-high and FAM111A-low LGG samples. Scale bar, 20µm.

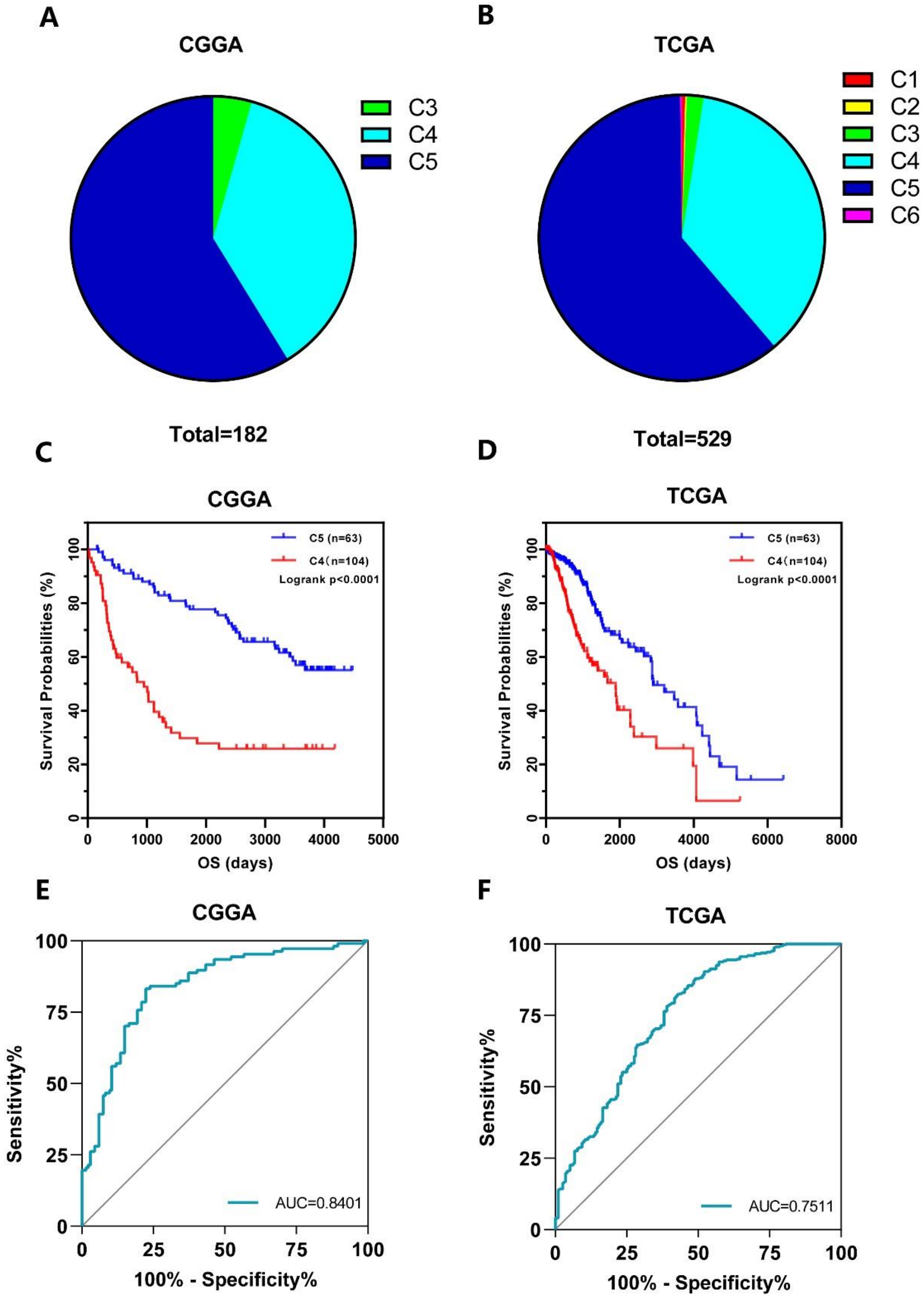

Supplementary Figure 5. The distribution of immune subtypes in the CGGA and TCGA cohort of lower-grade gliomas. (A, B) C4 and C5 consisted mostly of LGG. (C, D) Kaplan-Meier survival curves comparing the C4 and C5. Patients with C4 had the worse prognosis than C5 in lower-grade glioma in CGGA and TCGA datasets. (E, F) The predictive value of FAM111A expression for the C4 subtype in CGGA and TCGA dataset by ROC curve analysis.
